# A Case of Generalised Sarcoma, with Blood Changes

**Published:** 1907-12

**Authors:** F. G. Bushnell

**Affiliations:** Pathologist, Stephen Ralli Memorial, Brighton.


### A CASE OF GENERALISED SARCOMA, WITH BLOOD CHANGES.

BY

F. G. BUSHNELL, M.D.,

*Pathologist, Stephen Ralli Memorial, Brighton.*

---

JESSIE, æt. 21, under the care of Dr. Maynard. Patient was admitted on 24th September, 1906, for severe epistaxis and a hemorrhagic and purpuric eruption. There was a history of epistaxis occasionally for years. She was taken ill suddenly three weeks previous to admission with sickness and vomiting. On admission, there was considerable anæmia. There were enlarged, softish glands in the neck and axillæ. The splenic dulness extended to the costal margin, and the liver dulness from fourth rib to one finger's breadth below border of ribs. There was a hard, slightly movable, tender, nodular mass of tumour in the pelvis to each side of middle line, with a general tenseness of abdomen. The breasts were very tense, hard and nodular, and were said to have been so for three weeks. There were hemorrhagic purpuric spots on the arms and legs.

The temperature was 101.6° on admission. It rose on 26th September, 1906, to 106.2°, with a severe rigor. The patient died on the 27th September, 1906, at 2 a.m.

*Post-mortem findings* (by Dr. Nockald).—Malignant new growth of ovaries, retroperitoneal glands, mesentery, omentum, portal fissure, liver and breasts, peritoneal effusion, subcutaneous and petechial hemorrhages.

The thoracic viscera were not examined, permission being refused. For this reason also a limited examination of the bone-marrow could only be made.

The subject was a well nourished girl. There were numerous subcutaneous hemorrhages, especially on the outer side of the left leg, and on the inner side of the right leg. There was also a petechial eruption, most marked on the legs. On opening the abdomen, a moderate quantity of dark yellow fluid was found in the peritoneal cavity. The great omentum was matted together, and contained masses of what appeared to be new growth. These were diffuse, and on section had a pearly lustre and showed hemorrhages. The mesentery had undergone a similar change, which was generally most marked near the intestine. The iliac glands on the left side were not distinguishable as such, but their site was occupied by a large mass of growth similar to that in the omentum and mesentery, which reached from the fourth lumbar vertebra along the brim of the pelvis almost to the pubic bones.

The right ovary was large and nodulated, firm in consistence, and showed a blood cyst about two cm. diameter on the surface. It was not adherent to any of the surrounding structures. On section, the mass was solid, tough, and showed hemorrhages, and had all the appearance of a sarcoma. There were no naked-eye evidences of ovarian tissue. The left ovary was similar in appearance to the right, but only about half the size.

There were small masses of growth beneath the peritoneal covering of the uterus, particularly on the posterior surface. The cavity of the uterus was somewhat larger than normal. The vagina was normal, also the kidneys and supra-renal capsules.

The liver was rather pale ; the hilum contained masses of new growth. The gall-bladder was very thickened, apparently by inflammatory change (microscopically, this was seen to be sarcomatous). The spleen was slightly enlarged, but externally and on section was normal.

The breasts were firm, and contained numerous nodular, rather hard masses, which resembled new growth on section, and were hemorrhagic. Glandular breast tissue was seen between the nodules.

*Microscopic examination.*—The blood, ovaries, lymph glands, mesentery, oviduct, uterus, liver, gall-bladder, marrow and breasts were examined microscopically by ordinary (hæmatoxylin and eosin, Van Giesen) stains, by Pappenheim's pyronin methyl green, by Leishman's blood stain, and by eosin methylene blue stain in order to study the nature of the neoplasm and its relationship, if any, to inflammatory tissue, or to the lymphocytes of the blood. Micro-organisms, too, were searched for by Gram-Weigert's method of staining, but cultures of the blood were inadvertently not made before or after death.

The *ovaries* show polyhedral or rounded cells, with large nuclei and small amount of cytoplasm ; rarely they are swollen and dropsical. The latter usually is acidophilous. Between the cells are delicate fibrils ; in places the arrangement is alveolar. There are areas of degeneration and hemorrhages. In places there is a loose connective tissue with round or spindle cells, and red-blood cells are present. There are many thin-walled blood vessels.

*The breast.*—The fat or fibrous tissue is diffusely, and in places densely, infiltrated with large rounded cells resembling those in ovaries and is vascular, very necrotic and hemorrhagic.

*The liver.*—The portal fissure is the seat of a round-cell growth, which diffusely penetrates the liver and its capillaries ; the periportal tissue is infiltrated far away from the fissure.

*The mesentery and omentum* show growths of polygonal cells infiltrating the fat. The cells have large nuclei, with granules of chromatin and a thin rim of protoplasm.

*The walls of the gall-bladder* and the folds of its mucosa are infiltrated densely with round cells.

*The oviduct* (left near ampulla) has undergone sarcomatous change ; also the iliac glands.

There is sarcomatous tissue between the uterus and bladder, and diffusely throughout the pelvic viscera. The rib marrow was fatty.

Some of the cells are large, with two or more nuclei, but there are no large giant cells. As a rule, the cells are larger than mature lymphocytes.

No leucocytes or plasma cells appear.

The patient died three days after admission, and the day before death had 50 per cent. hæmoglobin, 2,960,000 red cells and 13,400 white cells, with a colour index of 0.83, and 1 white to 222 red cells. There was a marked relative lymphocytosis.

*Blood examination.*—Hæmoglobin, 50 per cent.; reds, 2,960,000; whites, 13,400; colour index, 0.83; white to red, 1 to 222. Polynuclears, 13.0; large lymphocytes, 37.3; small lymphocytes, 29.1; transitionals, 12.6; eosinophil polynuclears, 1; myelocytes, 3.4; large hyaline, 2.1.

This case is one of great interest, as it illustrates the close association of sarcomata and blood diseases. I have seen lymphocythæmia with deposits in the liver, kidney and marrow, and not markedly affecting the lymph glands; and again lymphocythæmia has been found with malignant mediastinal growths. Lymphadenoma has recently been described showing malignant (sarcomatous) infiltration of adjacent structures, and lymphosarcoma and sarcoma are found to occur with or without relative white cell changes in the blood. Daniels says that sarcomata always possess a stroma; otherwise lymphosarcoma is characterised by continuous infiltration, suggesting a direct infection by lymph paths, whereas sarcoma is said to form metastases more frequently. (The distinction of lymphosarcoma from Hodgkin's disease by the infiltration of the lung in the former can no longer be maintained.)

I would place our case among the sarcomata, though I admit that the various deposits were not proved to be truly "metastatic" beyond doubt. It is characterised by the marked "relative lymphocytosis" of the blood of the large cell type, by its hemorrhagic eruption, and its diffuse growth (as seen in the mesentery, omentum and portal fissure of liver). It is thus illustrative of the sarcomata which are at one end of the scale, of which lymphocythæmia is at the other. This supports the view taken by Banti that both myeloid and lymphatic leukæmias are true tumour developments, a sarcomatosis of medullary and lymphatic elements respectively. Banti believes a lymphocytosis may be mistaken for a discharge of sarcoma cells. It is suggested by Salaman that the clinical differences in these cases are physiological, and depend on the stage of development of the lymph-cell forming the tumours, in some cases resembling the finished lymphocyte; in others, and more malignant ones (as this of ours), resembling the lymphogonia of the germinal centres.

The sarcomatous nature of infective venereal tumours in dogs was described at the last meeting of the Pathological Society of Great Britain and Ireland, and has been confirmed by Seligmann. (They are similar to those described by Bellingham-Smith and Washbourne.) They are not the result of direct extension only, but may show true metastasis (*e.g.* into the testis). These tumours are then, as Hoch says, contagious malignant neoplasms, usually locally malignant. Our case showed no disease of vulva or vagina, and there is no evidence to place it in this category.

The connection between the sarcomatosis and the cutaneous hemorrhages is unknown. Engel Martens and Douglas obtained experimentally no specific precipitation of blood to cancer. Von Dungern, Beebe and Pearce, in the *Journal of Experimental Medicine* (vol. 7), have shown that hæmolytic and hæmagglutinative properties exist in a serum obtained by injection of various crushed tissues, mixed with blood, into an animal. It is possible such anti-bodies were circulating in this patient's blood; and if so, their presence was dependent on an affection of the blood itself.

Whether the blood was altered in its alkali content is not known. Neither did I find evidence of the phenomena observed by Farmer, Wallien, and Moore in the leucocytes in the active zone around early cancers. These indicated an interchange between the chromosomes of the daughter nuclei of the leucocytes and tissue cells after mitosis. No evidence of an infectious nature, or of "evolutionary" changes in the tissues, was observed. That Nature has some function for this apparently purposeless proliferation is certain.

A word may be said as to nomenclature of this disease. Warthin suggests "leucoblastomata"—based on Powell White's classification apparently—and "malignant lymphomata" or "multiple sarcomata," and "sarcomatosis" are suggested (among others) by some authorities.

In respect to the relation of lymphosarcoma to Hodgkin's disease, Gibbons records nine cases of Hodgkin's disease, with five autopsies, in which the blood picture was normal, and there were no relative changes in leucocytes. The glands showed the proliferation of the germ centres of follicles and endothelium of sinuses, and presence of giant cells, eosinophils, and a few plasma or polymorphonuclear cells, with changes in connective tissue structure, as described by Reed and Longcope, Simmons, and others. In the hard variety peculiar giant cells and small lymphocytes are striking features. The capsules *showed* infiltration, and even penetration (and incidentally the capsules were seen to be secondary envelopes). The muscle cells may be invaded, destroying them, or the submaxillary gland be invaded. The metastases present certain features of lymph glands; the boundaries usually are sharply defined, but without a capsule, and may infiltrate and destroy tissues around. In the spleen they may appear in Malpighian bodies, and stretch out as ill-defined nodules into the pulp.

The metastases in the lungs showed that they were not alone developments of pre-existing lymphoid tissue in organ, but malignant metastases pushing into adjacent tissue and destroying it. They occurred, too, in the lower lobe, and away from a bronchus.

The five autopsies of nine cases showed involvement of lymph glands all over the body, with metastases in liver in four, in spleen in four, in kidney in two, in lungs, pericardium and pancreas in one each. Of other four not coming to *post mortem*, one showed involvement of all the external glands.

In lymphadenoma Andrewes describes a loss of all germ centres, diminution of lymphocytes, increase of reticulum, and the presence of eosinophil cells and giant cells. Thus lymphocytes, lymphoid tissue, and connective tissue may be affected by a proliferation which can only be described as malignant, and lymphocytes, plasma cells and fibroblasts have been observed to be stages of growth by which wandering blood cells become fixed tissue cells.

